# Evaluation of Entomopathogenic Nematode Isolates against Subterranean Termites under Laboratory and Field Conditions

**DOI:** 10.12688/f1000research.138237.2

**Published:** 2024-02-22

**Authors:** Belay Abate Gutema, Dawit Melisie Achlehum, Tariku Tesfaye Edosa, Belay Feyisa, Fikremariam Yimer, Teshale Daba Dinka

**Affiliations:** 1 Agricultural Entomology, Ethiopian Institute of Agricultural Research, Ambo, Oromia, 37, Ethiopia; 2 Nematology, Ethiopian Institute of Agricultural Research, Ambo, Oromia, 37, Ethiopia

**Keywords:** Keywords: AEH, Damage, Isolate, Pathogenicity, S#50

## Abstract

**Background:**

Termites are a major insect pest affecting agricultural production and woody materials. They cause severe devastation in the ecosystem, and lead to bare soil. This phenomenon causes the soil to become difficult to plow, which in turn leads to a reduction in the productivity of crops. It can cause 100 % yield losses based on crop types, level of the damage, and size of its populations. To manage this pest, different management options have been evaluated in Ethiopia. While insecticide usage is the dominant option, less attention has been given to Entomopathogenic Nematode (EPN) based management options. Therefore, this research was initiated to screen locally collected EPN isolates and evaluate promising isolates under field conditions on maize crop.

**Methods:**

37 EPN isolates were screened under laboratory condition, while two isolates were evaluated at field condition. The screening of EPN isolates was laid out in a completely randomized design, and the field evaluation used a completely randomized block design, and treatments were replicated thrice. Mortality of insect, damaged root, stem, cob, damage severity, foraging termites, and yield of the crop data were collected.

**Results:**

The study indicated that all screened EPN isolates caused mortality on termites under laboratory conditions. The isolates achieved complete mortality of the insect pest within 12 days of exposure. The finding indicated that AEH and S#50 were the more pathogenic and virulent isolates on termites under laboratory conditions and taken to field study. The S#50 isolate was most pathogenic and reduced the infestation and severity of the insect pest on the maize crop under field conditions.

**Conclusions:**

This result showed that the entomopathogenic nematode isolates have the potential to manage subterranean termites in the maize field. Future studies should be based on collection of local isolates and develop a full package for the virulent isolates.

## Introduction

Termites are a major insect pest affecting agriculture in Ethiopia, especially in the western part of the country. In this area four termite generals from the Macrotermitinae subfamily are the dominant species affecting crop productions. Among them the Macrotermite general is the economically important insect pest in the country ( Abdurahaman, 1990). From 61 species recorded, 10 were confined to the country. *Macrotermes* sp. and *Microtermes* spp. were the major termites affecting agricultural production and woody structures in the country ( Cowie *et al*., 1990). Abdurahaman’s (1990) study showed that *Macrotermes subhyalinus* was the most common termite species found in Wollega and Assosa areas. The species was known to construct dome shaped mounds and was mostly observed by its foraging nature of the whole plant parts, crop stacks and woody litter lying on the soil surface.

Termites also cause severe devastation in the forest, leaving the soil bare and soil elements exposed to erosion ( Abraham, 1990; Kumar and Pardeshi, 2011; Bong *et al*., 2012). As a result, farmers are forced to abandon their farmland ( Abraham and Adane, 1995). Yield loss depends on the type of crop, the extent of the population, and the degree of termite infestation of the crop at different growth stages. Abdurahaman (1990) found that 45% removal of the crop at the six-leaf crop resulted in 16.5% yield loss, while the same reduction in the tassel stage resulted in 39.9% yield loss. But the severe infestations of termite species in Ethiopia can result in crop losses of up to 100% ( Nyeko *et al*., 2010).

Termites are most likely to target plants that are unfamiliar to the area and plants that are under water stress. Damage caused by these termites also provides access for secondary infections for pathogens, particularly aspergillus. Soil infested with termites usually leads to soil structure distortions and compaction. Therefore, the soil becomes difficult to plow, which in turn leads to a reduction in the productivity of crops ( Devendra *et al*., 1998; Hailemichael *et al*., 2013; Legesse *et al*., 2013).

Since the 1980s different termite management practices have been conducted to control termites in western Ethiopia. As an example, in 1983 the Ministry of Agriculture had poisoned 635,908 mounds by using Aldrin 40% WP insecticide in Menesibu and Nedjo-Jarso areas ( Abdurahaman, 1990). A similar method was used in Asosa and Anger Gutin on 2145 mounds which took 201 kg of the aforementioned product ( Abdurahaman, 1990). Additionally, 557,563 mounds were treated by 13,077 kg of Heptachlor 40% in Menesibu, Nedjo-Jarso, Ghimbi, Ayra-Guliso and Yubdo areas which was led by the Ministry of Coffee and Tea Development. On the other hand, more than 23,000 termite queens were collected as a management option of the pest during 1987-1988 in Ayra-Guliso area by the initiatives of Western Synod branch ( Abdurahaman, 1990).

Similarly, different plant botanicals have been evaluated for termite control in Ethiopia. As an example, neem seed extract was applied at 40 kg/ha and reduced the infestation of termite on hot pepper seedling in the Tanqua Abergelle district ( Gebreslasie and Meressa, 2018). Additionally, *Nicotiana tabacum* leaves and *Milletia ferruginea* seed extracts recorded complete mortality with 24 hours of the exposure time to termites under laboratory conditions. Whereas *Phytolacca dadecandra* leaf extracts achieved the same results after 48 hours, and *Azadirachta indica* leaves, *Hagenia abyssinica*, *Chrysanthemum* sp. and *Croton macrostachs* seed extracts recorded higher mortalities after 72 hours on the same insect and condition ( Tadele Shibiru *et al.,* 2013).

Even though termite management has been dominated by using persistent insecticides, it is not sustainable as a long-term solution and can affect biodiversity. Among the diverse potential alternatives available for termite management, the use of microbes is gaining prominence ( Michael, 2005). Entomopathogenic fungi, *Beauveria bassiana* and *Metarhizum anisophilae,* show promise for the management of various termite species in the laboratory ( Abebe, 2002; Milner, 2003; Addisu *et al*., 2014). However, reports of the use of entomopathogenic nematodes to control termites in Ethiopia are very limited and microbial-based treatment options have been limited to laboratory conditions. Although termites severely affect agricultural production in Ethiopia, an insufficient management option is available. Therefore, the study is initiated to screen locally collected entomopathogenic nematode isolates on termites under laboratory conditions and evaluate promising isolates under field condition on maize crop.

## Methods

### Ethics approval

This experiment was conducted within an appropriate ethical framework of Ethiopian Institute of Agricultural Research as confirmed by institutional letter with Ref. No.: 8.8/154/2023 written on 07 August 2023.

### Description of the study area

The laboratory experiment was conducted in the laboratory of the Entomology program at Ambo Agricultural Research Center, and the field evaluation was done at Bila district, East Wollega zone which is an insect pest-prone area. Termites, *Macrotermes* spp., were collected and established following Addisu *et al*. (2014) methods from Bako areas of the insect-prone maize field. Termite mounds were dug up using a spade, and soil containing termites was put on plastic sheets. The insects were collected from the plastic sheets using a camel-hair brush and placed in plastic boxes. Wooden plants (termite-infested materials) were added to the plastic boxes as feed for the termites. The top parts of the plastic boxes were covered with a mesh cloth that allowed air ventilation. A moistened wad of cotton was placed in the plastic boxes to maintain the moisture level for the survival of termites. The boxes carrying the termites were transported to the Ambo agricultural research center’s Entomology laboratory. Periodically, dry wooden materials were provided to the termite population, and the plastic boxes were inspected for maintenance of the required moisture level.

### Evaluation of EPN against termites under laboratory and field conditions

37 newly collected and preserved entomopathogenic nematode isolates were used for this experiment ( Table 1). Culturing and preparation of EPN were performed using standard methods described by Kaya and Stock (1997). Harvested nematodes were kept at 14°C in 250 ml flasks and used within a week of culture preparation. Each isolate was tested at 800 infective juveniles per ml concentrations and negative control (treated with sterile water) for comparison. 10 soldier termites were placed in a petri dish on filter paper and sprayed with the infective juvenile suspension. This study was conducted under laboratory conditions at 25 ± 2°C and 60% RH in dark places. The experiment was replicated three times and laid out in a completely randomized design.

**Table 1.** List of EPN isolates used for the experiment.

No.	EPN isolate code	EPN isolates Genus	Collection area	Origin
1	H.b	*Heterorhabditis bacteriophora*	South Africa	Soil
2	AEH	*Heterorhabditis* spp.	Ambo	Soil
3	HI	Strenematides spp.	Bule Hora	Soil
4	AW3	*Steinernema* spp.	Ambo	Soil
5	HH	Strenematides spp.	Bule Hora	Soil
6	Z9	*Heterorhabditis* spp.	Batu	Soil
7	S#20	Strenematides spp.	Adama	Soil
8	S#37	Strenematides spp.	Banja	Soil
9	S#43	*Heterorhabditis* spp.	Mecha	Soil
10	S#50	Strenematides spp.	Libo Kemkem	Soil
11	S#52	Strenematides spp.	Asossa	Soil
12	S#58	Strenematides spp.	Asossa	Soil
13	S#69	NA	NA	NA
14	S#72	*Heterorhabditis* spp.	Mana Sibu	Soil
15	S#74	*Heterorhabditis* spp.	Alamata	Soil
16	S#80	Strenematides spp.	Gobu Sayyo	Soil
17	J-01	Strenematides spp.	Jimma	Soil
18	APPRC-PL0631	*Steirnema* spp.	Jimma Zone	Soil
19	APPRC-PL0624	*Steirnema* spp.	Jimma Zone	Soil
20	APPRC-PL0612	*Heterorhabditis* spp.	Jimma Zone	Soil
21	S#19	*Heterorhabditis* spp.	Boset	Soil
22	APPRC-PL0056	*Steirnema* spp.	Wolenciti	Soil
23	APPRC-PL0697	*Heterorhabditis* spp.	Bako	Soil
24	APPRC-PL0508	*Steirnema* spp.	Jimma Zone	Soil
25	APPRC-PL0692	*Heterorhabditis* spp.	Bako	Soil
26	APPRC-PL0641	*Heterorhabditis* spp.	Jimma Zone	Soil
27	SY	*Steirnema yirgalemense*	Yirgalem, Ethiopia	Soil
28	S#67	*Heterorhabditis* spp.	Hintale Wojaret	Soil
29	S#59	*Heterorhabditis* spp.	Tahtay Koraro	Soil
30	S#70	Strenematides spp.	Adeigudem	Soil
31	S#76	Strenematides spp.	Bako	Soil
32	S#71	Strenematides spp.	Raya Azebo	Soil
33	S#76-1	NA	NA	Soil
34	S#2	NA	NA	Soil
35	S#72*	*Heterorhabditis* spp.	Raya Azebo	Soil
36	APPRC-PL0638	*Steirnema* spp.	Jimma Zone	Soil
37	S#8	*Heterorhabditis* spp.	Dufti	Soil

Note: NA – indicates the information is not available.

For the field experiment, Jibat variety of the crop used, and studied under natural infestation of termite. Two EPN (AEH and S#50) isolates were applied at a rate of 800 IJ/ml as a basal application at the seedling and tasseling growth stages of maize. Likewise, the recommended rate of diazinon at 60% EC (2.5 lit/ha) was applied. Untreated check plots were neither treated with the insecticide nor with isolates and recommended agronomic practices were applied. The plot size was 4 m long and 3 m wide, with intra-row spacing of 75 cm and inter-row spacing of 25 cm, with 2 m and 3 m spacing between plots and blocks, respectively. Two seeds per hill were sown, and then after emergence, the seedlings were thinned to adjust the population of the crop to one plant per hill. This is based on the maize production manuals of Ethiopia, which recommend 25 kg of maize seed per hectare ( Nigussie *et al.*, 2009). Data collection started two weeks after the first treatment application and continued every two weeks until physiological maturity of the crop. 10 plants were randomly sampled per plot to assess termite damage.

### Design, treatment arrangements, and data collection

The screening of EPN isolates was laid out in a completely randomized design, and the field evaluation used a completely randomized block design. Treatments were replicated three times in the experiments. AEH and S#50 isolates that recorded complete mortality of termites were considered promising isolates from laboratory results and taken to field conditions along with positive and negative checks. The following data were collected.

Damaged root: all plants in the plot were inspected for the termite damage on maize root at harvesting time, and plant roots that showed damage from termites were counted and expressed in percentages. Damaged stem: all plants in the plot were inspected for the termite damage on the maize stem, and plants that showed damage from termites were counted. Damaged cob: at physiological maturity, all cobs of maize in the plot were checked for damage by termites and counted. Damage severity: five maize plants were randomly taken from the plot, and a 0 – 9 scale were used to assess their damage. 0 indicates the plant was not attacked by termites, and 9 indicates that the plant was eaten and lodged to ground. Lodged plants: at physiological maturity, all maize plants in the plot were checked for lodging, and the numbers of plants lodged were counted. Foraging termites: all foraging termites observed in the plot were counted. Yield: at physiological maturity, maize cobs were harvested from each plot. The threshed grains moisture content was adjusted at 11 %, maize grains were weighed to determine the amount of yield, and the plot yields were converted to kg ha ^-1^. The insect and crop data collected were analyzed using statistical analysis software version 9.4 ( SAS, 2023). The least significant difference (LCD) at the 5% level was used to compare treatment means ( Gomez and Gomez, 1984).

## Results and discussion

### Screening of entomopathogenic nematodes in laboratory condition

All entomopathogenic nematode isolates screened caused mortality in termites under laboratory conditions ( Abate, 2023). These isolates showed significant differences among each other and with the negative control across the exposure time ( Table 1). 18% of tested isolates caused greater than 50% mortalities on the insect pest after the 3 ^rd^ day of the treatments’ application ( Table 1). On this date, S#50 and AEH isolates recorded higher mortalities at 70% and 63.33%, respectively, while the lower mortalities were from the APPRC-PL0056 isolate (20%) and negative control (10%). On the other hand, half of the tested isolates recorded greater than 50% termite mortalities after the 6 ^th^ day of the treatments’ application ( Table 2). But S#50 and AEH isolates achieved 90% and 80% mortality on the insect pest on the same date, respectively. 10.53% of isolates recorded greater than 75% of mortalities on insect pests after the 9 ^th^ day of their applications. Similarly, 92.11% of isolates caused greater than 50% of mortalities in the insect pest on this date. After the 12 ^th^ day of treatments applications, tested isolates showed significant differences with the negative control (p < 0.001) ( Table 1). On this date, AEH and S#50 isolates achieved complete mortality on the tested insect pest and showed significant differences with other. Similarly, 31.58 % and 94.74% of tested isolates recorded greater than 75% and 50% of mortalities on the insect pest, respectively. However, S#8 isolate recorded the minimum mortality (43.33%) among the entomopathogenic nematode isolates evaluated.

**Table 2.** Entomopathogenic nematode isolates screened on termite under laboratory condition.

EPN Isolates	Exposure days			
	After 3 ^rd^ days	After 6 ^th^ days	After 9 ^th^ days	After 12 ^th^ days
H.b	56.67 ^ab^	66.67 ^a-c^	66.67 ^a-c^	66.67 ^a-d^
AEH	63.33 ^ab^	80 ^ab^	93.33 ^a^	100 ^a^
HI	33.33 ^ab^	46.67 ^a-c^	63.33 ^a-c^	66.67 ^a-d^
AW3	43.33 ^ab^	60 ^a-c^	73.33 ^ab^	83.33 ^a-c^
HH	36.67 ^ab^	46.67 ^a-c^	60 ^a-c^	73.33 ^a-c^
Z9	50 ^ab^	53.33 ^a-c^	63.33 ^a-c^	80 ^a-c^
S#20	23.33 ^ab^	46.67 ^a-c^	66.67 ^a-c^	70 ^a-c^
S#37	56.67 ^ab^	73.33 ^ab^	83.33 ^ab^	86.67 ^ab^
S#43	43.33 ^ab^	60 ^a-c^	66.67 ^a-c^	70 ^a-c^
S#50	70 ^a^	90 ^a^	93.33 ^a^	100 ^a^
S#52	43.33 ^ab^	53.33 ^a-c^	66.67 ^a-c^	70 ^a-c^
S#58	43.33 ^ab^	60 ^a-c^	76.67 ^ab^	76.67 ^a-c^
S#69	50 ^ab^	60 ^a-c^	73.33 ^ab^	80 ^a-c^
S#72	33.33 ^ab^	43.33 ^a-c^	50 ^a-c^	53.33 ^b-d^
S#74	43.33 ^ab^	56.67 ^a-c^	70 ^a-c^	76.67 ^a-c^
S#80	36.67 ^ab^	56.67 ^a-c^	66.67 ^a-c^	80 ^a-c^
J-01	40 ^ab^	53.33 ^a-c^	70 ^a-c^	76.67 ^a-c^
APPRC-PL0631	30 ^ab^	40 ^a-c^	63.33 ^a-c^	70 ^a-c^
APPRC-PL0624	26.67 ^ab^	43.33 ^a-c^	63.33 ^a-c^	66.67 ^a-d^
APPRC-PL0612	36.67 ^ab^	56.67 ^a-c^	73.33 ^ab^	73.33 ^a-c^
S#19	33.33 ^ab^	46.67 ^a-c^	56.67 ^a-c^	73.33 ^a-c^
APPRC-PL0056	20 ^ab^	40 ^a-c^	53.33 ^a-c^	63.33 ^a-d^
APPRC-PL0697	36.67 ^ab^	56.67 ^a-c^	73.33 ^ab^	83.33 ^a-c^
APPRC-PL0508	33.33 ^ab^	53.33 ^a-c^	70 ^a-c^	80 ^a-c^
APPRC-PL0692	23.33 ^ab^	33.33 ^a-c^	43.33 ^a-c^	70 ^a-c^
APPRC-PL0641	43.33 ^ab^	53.33 ^a-c^	63.33 ^a-c^	70 ^a-c^
S.Y	40 ^ab^	53.33 ^a-c^	60 ^a-c^	63.33 ^a-d^
S#67	43.33 ^ab^	43.33 ^a-c^	50 ^a-c^	53.33 ^b-d^
S#59	53.33 ^ab^	60 ^a-c^	66.67 ^a-c^	66.67 ^a-d^
S#70	36.67 ^ab^	46.67 ^a-c^	56.67 ^a-c^	60 ^a-d^
S#76	40 ^ab^	50 ^a-c^	56.67 ^a-c^	56.67 ^b-d^
S#71	46.67 ^ab^	53.33 ^a-c^	56.67 ^a-c^	63.33 ^a-d^
S#76-1	33.33 ^ab^	40 ^a-c^	50 ^a-c^	60 ^a-d^
S#2	43.33 ^ab^	46.67 ^a-c^	56.67 ^a-c^	56.67 ^b-d^
S#72*	43.33 ^ab^	50 ^a-c^	56.67 ^a-c^	56.67 ^b-d^
APPRC-PL0638	40 ^ab^	46.67 ^a-c^	56.67 ^a-c^	56.67 ^b-d^
S#8	26.67 ^ab^	30 ^bc^	40 ^bc^	43.33 ^cd^
Negative control	10 ^b^	10 ^c^	20 ^c^	26.67 ^d^

Note: Means with the same letters within the column are not significantly different at 0.05 probability level.

This result revealed that the tested entomopathogenic nematode isolates were pathogenic to the termites and caused mortality. The effectiveness of all isolates showed an increasing trend across the exposure durations. Some isolates achieved complete mortality of the insect pest within 12 days of exposure. The finding indicated that AEH and S#50 were the more pathogenic and virulent isolates on termites under laboratory conditions. Based on this result, AEH and S#50 isolates were considered as promising under field conditions. Similarly, microbials bioagent ( *Metarhizium anisoplie* and *Beauveria bassiana*) had showed promising results under laboratory conditions for Macrotemes spp management ( Addisu *et al*., 2014). Entomopathogenic nematodes (EPNs) are helpful nematodes that have a high potential for controlling insects in soil environments and are commercially employed to treat a wide range of pests ( Verma *et al*., 2018). In a filter paper and sand test, Shahina and Tabassum (2010) found that EPN produced increased mortalities in the subterranean termite. Yu *et al*. (2010) investigated the pathogenicity of three new strains of *Steinernema riobrave* (3-8b, 7-12, and TP) against workers of *Heterotermes aureus*, *Reticulitermes flavipes*, and *Coptotermes formosanus.* *Heterotermes aureus* was shown the most sensitive to all *S. riobrave* strains, and termites in all nematode treatments died after four days. The TP strain caused 75% and 91% mortality in *R. flavipes* and *C. formosanus*, respectively. More research was suggested to determine *S. riobrave* (TP)’s potential to control the targeted termite species in the field condition ( Yu *et al*., 2010).

### Evaluation of promising entomopathogenic nematode isolates under field condition

The effectiveness of AEH and S#50 entomopathogenic nematode isolates showed a non-significant difference in the number of foraging termites and maize root damage in maize fields ( Table 3). However, treatments indicated a significant difference in stem damage, lodged plant, cob damage, the severity of insect damage, and yield (p < 0.001) ( Table 3). The lower stem damage was recorded by the S#50 isolate and positive control, while the higher stem damage was recorded by the AEH isolate. Similarly, the AEH isolate and negative control (untreated plot) showed higher cob damage and lodged plants and were more severely attacked by the insect pest, whereas S#50 and positive control showed significant differences, and lower lodged plants and cobs were damaged. Similarly, negative control and AEH isolates gave a lower maize yield (2111 kg/ha), while the highest yield was obtained from positive control ( Table 3).

**Table 3.** Effectiveness of EPN isolates on maize under field condition.

Treatments	Foraging termite	Stem damaged (mean)	Root damaged (%)	Lodged plants (mean)	Cob damaged (mean)	Severity (0-9)	Yield (kg/ha)
**AEH**	1.33(1.27)	17 ^a^	83.33	18.33 ^a^	2.67 ^a^	6.67 ^a^	2111 ^c^
**S#50**	2.67(1.74)	12 ^b^	94.44	12.33 ^b^	1 ^b^	5 ^ab^	2444.33 ^b^
**Diazinon**	0.33(0.88)	12.33 ^ab^	77.78	12.33 ^b^	1 ^b^	3 ^b^	2944.33 ^a^
**Control**	2.33(1.68)	14.33 ^a^	94.44	17.67 ^a^	3 ^a^	6.67 ^a^	2111 ^c^
**LSD**	0.82	2.03	ns	3.92	0.54	2.24	223.19
**CV**	29.75	7.76	21.03	13.73	15.06	22.32	4.9

The experiment indicated that the S#50 entomopathogenic nematode isolate showed a non-significant difference with the positive control on all considered parameters except yield data. This revealed that the isolate is more pathogenic and reduced the infestation and severity of the insect pest on the maize crop under field conditions. Additionally, the S#50 isolate increased the maize yield by 13.63% over the negative control. This result showed that the entomopathogenic nematode isolates have the potential to manage termites in the maize field. Subterranean termites’ dwell and forage in damp, cool, and shaded environments such as soil or wood materials. These characteristics are favorable for the survival and mobility of nematodes which enhances their role in termite management. Wang *et al*. (2002) and Yu *et al*.’s (2006) studies showed that entomopathogenic nematodes have the potential to be used as an environmentally safe alternative termite control method. *Steinernema* spp. and *Heterohabditis* spp. provided some limited control of subterranean termites in field studies but were largely ineffective for long-term suppression ( Mauldin and Beal, 1989). However, entomopathogenic nematodes have been used in classical, conservation, and augmentative biological control strategies. In addition to other nuisance insects, commercially generated EPNs are being used to manage scarab larvae in lawns and turf, fungus gnats in mushroom production, invasive mole crickets in lawns and turf, black vine weevils in nursery plants, and Diaprepes root weevils in citrus ( Lacey and Georgis, 2012). Similarly, Aslam *et al*.’s (2023) study indicated that *Steinernema carpocapsae*, *Heterorhabditis bacteriophora* and *Heterorhabditis indica* species recorded higher mortality of termites under field conditions. As a result, they suggested that indigenous EPNs can provide more effective termite control, presumably due to their direct interaction with pest species in the soil and the likelihood of secondary infection via infected cadavers.

## Conclusion

All screened entomopathogenic nematode isolates caused mortalities on termite under laboratory condition, and their pathogenicity was varied among isolates. The study showed that the S#50 and AEH isolates were more virulent than others and achieved complete mortalities on the tested insect pests within 12 days of the exposure time. As a result, these isolates were considered as promising isolates and tested under field condition. Similarly, S#50 isolate showed lower stem damage, lodged plant, cob damage, and severity of insect damage on the maize under field condition. From this experiment it can be concluded that entomopathogenic nematodes have a potential for termite management and part of integrated the insect pest management. Therefore, future studies should be focused on the collection of entomopathogenic nematodes from the insect cadaver at insect pest probe areas, screening, evaluating and considering it integrated in the insect pest management components, and develop a full package product for the promised isolates.

## Data Availability

figshare: EPN dataset. https://doi.org/10.6084/m9.figshare.24208092.v1 ( Abate, 2023). Data are available under the terms of the Creative Commons Attribution 4.0 International license (CC-BY 4.0). ARRIVE checklist for ‘Evaluation of entomopathogenic nematode isolates against subterranean termites under laboratory and field conditions’. https://doi.org/10.6084/m9.figshare.24526885.v1
