# Supplementary material for: Evaluation of Entomopathogenic Nematode Isolates against Subterranean Termites under Laboratory and Field Conditions
Source: F1000Res. 2025 Jan 3;12:1601. Originally published 2023 Dec 19. [Version 3] doi: 10.12688/f1000research.138237.3 (PMC11126895; doi:10.12688/f1000research.138237.3)
Supplement: Peer review history

# Reviewer report: Reviewer response for version 3

**DOI:** 10.5256/f1000research.174670.r355845

**Published:** 2025-01-25

**Authors:** Yaw Danso

**Affiliations:** 1 CSIR-Crops Research Institute, Kumasi, Ghana

Scientifically, one does not start a sentence (s) with figures but words.  

 KEYWORDS: Give proper definition to isolates AEH and S#50. 

 INTRODUCTION: Did you mean to write genera or generals relating to the termite classification?

 METHODS: Did the collection point identify the EPN collections or did the authors take the opportunity to identify them? In Table 1, educate me on the Strenematids sp. as EPNs.  Did you want to write Steinernema sp. or what? Check again. 'Juveniles' 

 Is it convention that intra-row spacing be higher or wider than inter-row? 75 cm and 25 cm, respectively. I am sure it is the vice versa. Do not start sentences with figures (10 plants). Consistency is key in scientific writing. In one part, you indicated that 10 plants per plot were assessed. in another part, all plants in a plot were assessed. 

 REFERENCES: Check and see below if the following references have been cited in the text;

 - Shiberu et al.

 - Shilesi et al. 

 - Sisay et al. 

 - Yu et al. 

 and 

 - Legese et al.

Is the work clearly and accurately presented and does it cite the current literature?

Yes

If applicable, is the statistical analysis and its interpretation appropriate?

Yes

Are all the source data underlying the results available to ensure full reproducibility?

Yes

Is the study design appropriate and is the work technically sound?

Yes

Are the conclusions drawn adequately supported by the results?

Yes

Are sufficient details of methods and analysis provided to allow replication by others?

Yes

Reviewer Expertise:

Nematology; Entomopathogenic Nematology

I confirm that I have read this submission and believe that I have an appropriate level of expertise to confirm that it is of an acceptable scientific standard.

# Reviewer report: Reviewer response for version 3

**DOI:** 10.5256/f1000research.174670.r358356

**Published:** 2025-01-21

**Authors:** El-Sayed H Shaurub

**Affiliations:** 1 Cairo University, Giza, Egypt

The authors have screened locally collected EPN isolates and evaluated their virulence against subterranean termites under laboratory and field conditions on maize crop. The authors have carried out a lot of experiments. The paper is good, well- designed and revealed helpful data. There are some points that need to be addressed as follows:

**Keywords**

 They are not informative. They lack informative keywords, such as entomopathogenic nematodes, virulence, subterranean termites, host plant damage.

**Title**

 Change the title to:

 Evaluation of the Effectiveness of Entomopathogenic Nematode Isolates against Subterranean Termites under Laboratory and Field Conditions

**Introduction**

**-**The 5 ^th^ and 6 ^th^ paragraphs are irrelevant to the current study as they focused on the use of botanicals and entomopathogenic fungi (EPF) to manage the subterranean termite, respectively.

**Methods**

**-**Please, place the statistical analysis as a separate subheading, instead of embedding them within the Materials and Methods.

 -Move the reasons of choosing the isolates AEH and S # 50, for the purpose of field experiments, from page 6, 1 ^st^ paragraph, last sentence, subheading “Experimental designs and data collections” to page 5, 3 ^rd^ sentence, subheading “Evaluation of EPN against termites under field conditions”.

 -Provide the day, month, and year during which the field experiments were carried out.

 -Provide the physicochemical characteristics of the soil (temperature, humidity and texture).

 -The authors did not give the reasons of using diazinon. Is it a chemical standard for the comparison with EPNs or what?

 -In the last sentence of materials & Methods, please replace the abbreviation of the Least Significant Difference, “LCD”, by LSD.

**Results and Discussion**

 -Page 6, subheading ** “**Screening of entomopathogenic nematodes in laboratory condition”, please replace Table 1 by Table 2.

 -Table 2, the 1 ^st^ heading “EPN Isolates”, please replace by “EPN Isolate Code” for similarity with the 2 ^nd^ heading of Table 1.

 -The mode of action of EPF is different from that of EPNs. Therefore, EPF are unfitting to discuss EPF.

 -Data of diazinon (Table 3) were missing in the Results.

Is the work clearly and accurately presented and does it cite the current literature?

Yes

If applicable, is the statistical analysis and its interpretation appropriate?

Yes

Are all the source data underlying the results available to ensure full reproducibility?

Yes

Is the study design appropriate and is the work technically sound?

Yes

Are the conclusions drawn adequately supported by the results?

Yes

Are sufficient details of methods and analysis provided to allow replication by others?

Yes

Reviewer Expertise:

Pesticide biochemistry and physiology, Insect control, Toxicology, Insect immunology, Insect histology

I confirm that I have read this submission and believe that I have an appropriate level of expertise to confirm that it is of an acceptable scientific standard, however I have significant reservations, as outlined above.

# Reviewer report: Reviewer response for version 3

**DOI:** 10.5256/f1000research.174670.r355846

**Published:** 2025-01-21

**Authors:** Gerald Medina Duza

**Affiliations:** 1 Cagayan State University- Piat Campus, Cagayan, Philippines

1. A systematic breakdown of results and discussion in separate sections would improve readability.

 2. More detail is required regarding the timing and method of entomopathogenic nematode (EPN) collection, the specific field conditions, and how termite populations were managed during field trials.

 3. Specify statistical tests used.

 4. Conclusions could benefit from a more balanced discussion about potential limitation such as climatic factors, and longer-term effects of EPN in field conditions.

Is the work clearly and accurately presented and does it cite the current literature?

Yes

If applicable, is the statistical analysis and its interpretation appropriate?

Yes

Are all the source data underlying the results available to ensure full reproducibility?

Yes

Is the study design appropriate and is the work technically sound?

Yes

Are the conclusions drawn adequately supported by the results?

Yes

Are sufficient details of methods and analysis provided to allow replication by others?

Yes

Reviewer Expertise:

Entomology- Insect Pathology and Microbiology

I confirm that I have read this submission and believe that I have an appropriate level of expertise to confirm that it is of an acceptable scientific standard.

# Reviewer report: Reviewer response for version 2

**DOI:** 10.5256/f1000research.162836.r276303

**Published:** 2024-07-24

**Authors:** Pamela Bruno

**Affiliations:** 1 Plant Production Systems, Agroscope, Zürich, Switzerland

This manuscript shows an extensive study of EPN collection, identification and evaluation of the collected EPN to control termites, carried out both in laboratory and field conditions.

 The project is very interesting and the experiments were well conducted, but the overall writing of the manuscript should be revised using better scientific language and following the scientific method.

 Some specific comments:

 Please describe with more detail the EPN collection (when?), rearing and identification (see literature as example).

 The lab experiment and the field experiment should be explained separately in the materials and methods, and instead of adding a separate title for "Design, treatment arrangements and data collection", it would be easier to understand each experiment if the information was all together under "Evaluation of EPN against termites under laboratory conditions" and "Evaluation of EPN against termites on field conditions".

 Author guidelines for this journal suggest to write results and discussions separately. This would help the reader better understand the paper.

 For the statistics, please provide more data about which tests were conducted (ANOVA, which type?...).

  Check grammar.

Is the work clearly and accurately presented and does it cite the current literature?

Partly

If applicable, is the statistical analysis and its interpretation appropriate?

Partly

Are all the source data underlying the results available to ensure full reproducibility?

No

Is the study design appropriate and is the work technically sound?

Yes

Are the conclusions drawn adequately supported by the results?

Partly

Are sufficient details of methods and analysis provided to allow replication by others?

Partly

Reviewer Expertise:

insect chemical ecology, tritrophic interactions, EPN, biological control

I confirm that I have read this submission and believe that I have an appropriate level of expertise to confirm that it is of an acceptable scientific standard, however I have significant reservations, as outlined above.

# Response

**Published:** 2024-11-13

**Authors:** Belay Abate

**Affiliations:** Agricultural Entomology, Ethiopian Institute of Agricultura Research, Ambo, Oromia, Ethiopia

Authors responses to Reviewers suggestions:

- Reviewers’ suggestion: overall writing of the manuscript should be revised using better scientific language and following the scientific method.
- Authors response: Based on reviewers’ suggestion we made revisions on the grammatical corrections as much as possible.
- Reviewers’ suggestion: Please describe with more detail the EPN collection (when?), rearing and identification (see literature as example).
- Authors response: The EPN isolates collection year were clearly indicated, and related information filled in the table 1. The EPN isolates were multiplied by using *Galleria mellonella* larvae, host insect to obtain infective juvenile following Kaya and Stock (1997)  procedures. And identification of EPN isolates done as per Tesfaye *et al*. (2021) procedures.
- Reviewers’ suggestion: The lab experiment and the field experiment should be explained separately in the materials and methods, and instead of adding a separate title for "Design, treatment arrangements and data collection", it would be easier to understand each experiment if the information was all together under "Evaluation of EPN against termites under laboratory conditions" and "Evaluation of EPN against termites on field conditions".
- Authors response: Laboratory and field experiments are separated into independent sub-title under methodology part. However, discussion part is presented under each topic (laboratory and field experiment).
- Reviewers’ suggestion: Author guidelines for this journal suggest to write results and discussions separately. This would help the reader better understand the paper.
- Authors response: It is possible to put the discussion in separate and at the end of the result. But we believed that it is better to put it under each sub-title for more coherence and attraction.  Finally, as much as possible we tried and followed the authors guideline of the F1000 journal publisher.
- Reviewers’ suggestion: For the statistics, please provide more data about which tests were conducted (ANOVA, which type?...).
- Authors response: The insect and crop data collected were analyzed using statistical analysis software version 9.4. The least significant difference (LCD) at the 5% level was used to compare treatment means. 

# Reviewer report: Reviewer response for version 2

**DOI:** 10.5256/f1000research.162836.r276302

**Published:** 2024-05-24

**Authors:** Eustachio Tarasco

**Affiliations:** 1 Department of Soil, Plant and Food Sciences, University of Bari “Aldo Moro”, Bari, Italy

Evaluation of Entomopathogenic Nematode Isolates against Subterranean Termites under Laboratory and Field Conditions is and Interesting paper quite well written and organized with something to be corrected above all on systematics. I recommended minor revision. And the related notes and correction are reported on the attached file. Anyway the main things to revise are of systematic nature.

Is the work clearly and accurately presented and does it cite the current literature?

Yes

If applicable, is the statistical analysis and its interpretation appropriate?

Yes

Are all the source data underlying the results available to ensure full reproducibility?

Yes

Is the study design appropriate and is the work technically sound?

Yes

Are the conclusions drawn adequately supported by the results?

Yes

Are sufficient details of methods and analysis provided to allow replication by others?

Yes

Reviewer Expertise:

entomology & nematodllogy

I confirm that I have read this submission and believe that I have an appropriate level of expertise to confirm that it is of an acceptable scientific standard.

# Reviewer report: Reviewer response for version 2

**DOI:** 10.5256/f1000research.162836.r249247

**Published:** 2024-03-12

**Authors:** Preety Tomar

**Affiliations:** 1 Department of Zoology, Akal College of Basic Sciences, Eternal University, Baru Sahib, Himachal Pradesh, India

No Comments

Is the work clearly and accurately presented and does it cite the current literature?

Yes

If applicable, is the statistical analysis and its interpretation appropriate?

Partly

Are all the source data underlying the results available to ensure full reproducibility?

Yes

Is the study design appropriate and is the work technically sound?

Yes

Are the conclusions drawn adequately supported by the results?

Yes

Are sufficient details of methods and analysis provided to allow replication by others?

Yes

Reviewer Expertise:

Entomopathogenic nematology, Biocontrol, Microbiology

I confirm that I have read this submission and believe that I have an appropriate level of expertise to confirm that it is of an acceptable scientific standard.

# Reviewer report: Reviewer response for version 1

**DOI:** 10.5256/f1000research.151421.r236149

**Published:** 2024-02-07

**Authors:** Preety Tomar

**Affiliations:** 1 Department of Zoology, Akal College of Basic Sciences, Eternal University, Baru Sahib, Himachal Pradesh, India

The research article entitled "Evaluation of Entomopathogenic Nematode Isolates against Subterranean Termites under Laboratory and Field Conditions" is very impressive work conducted by the authors. The article gives the complete information about the problems due to termite infestation and their management strategies. The results of the present investigation are highlighting the bio-control potential of entomopathogenic nematode isolates against termites not only in the laboratory but also under field conditions. However, its a suggestion that do mention which statistical analysis you have applied to compile the data and formulated the results. 

 Also add more information about the maize sowing and how you placed and distribute the termites over these plants under field conditions.

Is the work clearly and accurately presented and does it cite the current literature?

Yes

If applicable, is the statistical analysis and its interpretation appropriate?

Partly

Are all the source data underlying the results available to ensure full reproducibility?

Yes

Is the study design appropriate and is the work technically sound?

Yes

Are the conclusions drawn adequately supported by the results?

Yes

Are sufficient details of methods and analysis provided to allow replication by others?

Yes

Reviewer Expertise:

Entomopathogenic nematology, Biocontrol, Microbiology

I confirm that I have read this submission and believe that I have an appropriate level of expertise to confirm that it is of an acceptable scientific standard, however I have significant reservations, as outlined above.

# Response

**Published:** 2024-02-17

**Authors:** Belay Abate

**Affiliations:** Agricultural Entomology, Ethiopian Institute of Agricultura Research, Ambo, Oromia, Ethiopia

Based on the reviewers’ suggestions, minor revisions are made to the methodology and reference. In the methodology part, the collection site of SY isolate is Yirgalem, Ethiopia. Additionally, the insect and crop data collected were analyzed using statistical analysis software version 9.4. The least significant difference (LCD) at the 5% level was used to compare treatment means. 

 For the field experiment, the Jibat variety of the crop was used and studied under the natural infestation of termites. Two seeds per hill were sown, and then, after emergence, the seedlings were thinned to adjust the population of the crop to one plant per hill. This is based on the maize production manuals of Ethiopia, which recommend 25 kg of maize seed per hectare. And the revision is also done for reference.
